# Mortality Trends Observed in Population-Based Surveillance of an Urban Slum Settlement, Kibera, Kenya, 2007–2010

**DOI:** 10.1371/journal.pone.0085913

**Published:** 2014-01-28

**Authors:** Beatrice Olack, Daniel R. Feikin, Leonard O. Cosmas, Kennedy O. Odero, George O. Okoth, Joel M. Montgomery, Robert F. Breiman

**Affiliations:** 1 Kenya Medical Research Institute and Centers for Disease Control and Prevention Collaboration (KEMRI-CDC), Nairobi, Kenya; 2 Global Disease Detection Division, Kenya Office of the US Centers for Disease Control & Prevention, Nairobi, Kenya; Vanderbilt University, United States of America

## Abstract

**Background:**

We used population based infectious disease surveillance to characterize mortality rates in residents of an urban slum in Kenya.

**Methods:**

We analyzed biweekly household visit data collected two weeks before death for 749 cases who died during January 1, 2007 to December 31, 2010. We also selected controls matched by age, gender and having a biweekly household visit within two weeks before death of the corresponding case and compared the symptoms reported.

**Results:**

The overall mortality rate was 6.3 per 1,000 person years of observation (PYO) (females: 5.7; males: 6.8). Infant mortality rate was 50.2 per 1000 PYOs, and it was 15.1 per 1,000 PYOs for children <5 years old. Poisson regression indicates a significant decrease over time in overall mortality from (6.0 in 2007 to 4.0 in 2010 per 1000 PYOs; p<0.05) in persons ≥5 years old. This decrease was predominant in females (7.8 to 5.7 per 1000 PYOs; p<0.05). Two weeks before death, significantly higher prevalence for cough (OR = 4.7 [95% CI: 3.7–5.9]), fever (OR = 8.1 [95% CI: 6.1–10.7]), and diarrhea (OR = 9.1 [95% CI: 6.4–13.2]) were reported among participants who died (cases) when compared to participants who did not die (controls). Diarrhea followed by fever were independently associated with deaths (OR = 14.4 [95% CI: 7.1–29.2]), and (OR = 11.4 [95% CI: 6.7–19.4]) respectively.

**Conclusions:**

Despite accessible health care, mortality rates are high among people living in this urban slum; infectious disease syndromes appear to be linked to a substantial proportion of deaths. Rapid urbanization poses an increasing challenge in national efforts to improve health outcomes, including reducing childhood mortality rates. Targeting impoverished people in urban slums with effective interventions such as water and sanitation interventions are needed to achieve national objectives for health.

## Introduction

Africa is urbanizing at a faster rate than any other continent in the world [1]. By 2030, more than half of the population of sub-Saharan Africa is expected to live in urban areas [2]. More than 70% of urban residents in sub-Saharan Africa live in informal settlements or slums [3], where basic infrastructure cannot keep pace with urbanization rates [4]. These slums are characterized by dense population, poor sanitation, and contaminated water supplies, with potential adverse health impact for community residents [5], [6]. Consequently, such slums are often characterized by high incidence of a variety of communicable and non-communicable diseases [7] and injuries, leading to high rates of morbidity [8] and alarmingly high under-five mortality rates [9].

Mortality rate information is crucial for setting priorities for health interventions and research [10]. While recent studies have examined childhood mortality rates in rural or peri-urban areas [11], [12]. little is known about mortality in children and adults residing in informal urban settlements in Africa. Furthermore, data available on health status are often derived from hospital-based studies that neglect morbidity and mortality outside of health care systems accessed by a minority of people in African urban and rural settings [13], [14]. likely underestimating disease burden. In this paper, we describe the overall and age-specific mortality rates and trends over time in a well-defined population in Kibera, an urban slum in Nairobi, Kenya, by utilizing population-based surveillance data collected from January 2007 to December 2010.

## Materials and Methods

The Kenya Medical Research Institute (KEMRI) and the US Centers for Disease Control and Prevention (CDC) have collaboratively conducted population-based infectious disease surveillance (PBIDS) since late 2005 in 2 villages (Gatwikera and Soweto West) in the Kibera slum, situated in Nairobi, Kenya. Population ranges between 25,000 and 29,000 people in the 0.37 km^2^ surveillance area (mean population density = 77,000 persons/km^2^). Consistent with conditions in most urban slums, the PBIDS site is characterized by sub-standard housing and poor security, insufficient garbage disposal, lack of formal sewers, overcrowding and inadequate water drainage.

PBIDS has been previously described [8]. Standardized home visit data were captured electronically with Personal Digital Assistants (PDA) by trained community interviewers who visited about 7000 enrolled households within the surveillance villages of Gatwikira and Soweto West every two weeks, asking standardized questions about illnesses and specific symptoms for each person in the household. Morbidity and vital events data were recorded for each person in the household. In the event of death, a household proxy was interviewed to collect relevant information about illnesses and health-seeking behaviour before death. Ill participants enrolled in the study could access free high quality care at a well staffed study clinic run in collaboration between Carolina for Kibera and KEMRI/CDC and located within 1 km of the surveillance area residence. Data used for the current analysis were abstracted from the PBIDS database for the years 2007–2010 (the study period). All participants registered into PBIDS were eligible for the study.

To be enrolled into PBIDS, participants must have resided in the area for at least 4 consecutive calendar months, and the household head consented to participation. Residency status in the surveillance area was used to determine start and stop dates for person-time (rate denominator) contribution. The difference between the start and stop dates were divided by 365.25 to generate person years contributed to the study [15].

Symptoms and care seeking behaviour occurring two weeks before death were determined from household interview data. Case definition for fever and diarrhoea symptoms are as earlier defined [13]. We used local language (Kiswahili or Dho-luo) terms when asking about presence of cough during the biweekly interviews.

Data analyses were performed using SAS (Statistical Analysis System, version 9.3, Cary, NC). A post hoc case control analysis was undertaken to determine the symptoms associated with death. Mortality rates were expressed as the number of deaths (overall or by age-group) of participants per 1,000 person years of observation (PYO) [15]. We defined infant mortality as deaths in children <12 months old, child mortality as deaths in children 12–59 months and under-five mortality rate as deaths in all children less than 60 months.

We compared age-group associated mortality rates using incidence rate ratios and used poisson regression to model for linear trend in the mortality rates. Pair-Matched Odds Ratio was used to evaluate whether an association exists between case status (for cases and controls) and death outcome. We used multivariate conditional logistic regression to evaluate whether illnesses reported by cases and controls differed and to determine which illness or group of illnesses are best predictors of the outcome variable (case status). Two and three way interactions were fitted and their significance tested using p-values. All insignificant interactions were dropped to obtain a well-fitting model.

Cases were all participants in PBIDS who died between January 2007 and December 2010 and had pre- mortem information from the biweekly household visit. Controls were participants who were alive at the time that their respective cases died. We selected age ranges which provided at least two controls per case with a target of three controls per case matched by gender, age and a biweekly household interview within 2-weeks before death of the corresponding case. To select controls, we used the following age ranges matched to each age category of cases: for each case <12 months old, matching controls were plus or minus 2 months of age around the age of the matching case; for each case 1-to-4 year of age, matching controls were within plus or minus 2 months of age; for each case aged 5 to 17 years old, matching controls were plus or minus 1 year of age, for each case aged 18 to 34 years old, matching controls were plus or minus 2 years of age. In selecting the controls for each case >35 years old, matching controls were plus or minus 3 years of age.

Data collection was conducted as part of the KEMRI/CDC PBIDS protocol. The PBIDS protocol has been reviewed and approved by the Ethical Review Boards of the Kenya Medical Research Institute (KEMRI) (Protocol #932 and #1899) and the CDC's Institutional Review Board (#4566). Trained community interviewers obtained written informed consent for participants in the PBIDS study. For minors, consent was obtained from their parent or guardian.

## Results

During the study period, PBIDS data comprised 46,022 individuals accumulating 119,119 person years. Children under five years contributed 17.2% of the total person time (Table 1). A total of 749 deaths were recorded during the study period, yielding an overall mortality rate of 6.3 (95% CI: 5.9, 6.8) per 1000 PYO. The mortality rate for children <5 years old, 15.1 (95% CI: 13.5, 16.9) per 1,000 PYO, was more than three-fold that for persons ≥5 years old (4.5 [95% CI: 4.1, 4.9] per 1,000 PYO). Half of the deaths (52%) in children under five were among infants. The infant mortality rate (50.2 [95% CI: 43.0, 58.6] per 1000 PYO) was >5-fold higher than the child mortality rate (8.6 [95% CI: 7.3, 10.1] per 1000 PYO).

**Table 1:** Characteristics of the study population.

Variable	PYO	Percentage
**All**	119119	
**Sex**		
Male	60647	50.9
Female	58472	49.1
**Age in years**		
<1	3210	2.7
1–4	17270	14.5
5–9	18035	15.1
10–17	18474	15.5
18–34	44784	37.6
35–49	14134	11.9
≥50	3211	2.7
**Year of observation**		
2007	28798	24.2
2008	29542	24.8
2009	30054	25.2
2010	30725	25.8

There was a significant decline in overall annual mortality rates (7.7 in 2007 to 6.0 in 2010 per 1000 PYOs; p<0.05) over the four years. However, when stratified by age group, significant reductions over time were only shown for adults 18–34 year old (Table 2). The decline (p<0.05) in this age category was in both males and females. Over the course of the study period, a significant decline (p<0.05) in mortality rates was observed among all females regardless of age-but not among males (Table 2).

**Table 2:** Trend in mortality rates by gender, age and year in Kibera informal settlement, Kenya January 2009–December 2010.

	2007	2008	2009	2010	Overall	P-value
**Sex**						
Male	7.6	6.4	6.3	6.9	6.8	0.47
Female	7.8	5.5	4.8	5.0	5.7	<0.05*
**Age in years**						
<1	51.1	35.5	49.3	63.9	50.2	0.14
1–4	9.4	10.0	8.0	6.8	8.6	0.13
5–9	1.4	0.9	1.3	2.3	1.5	0.22
10–17	1.7	0.5	1.1	0.8	1.0	0.33
18–34	6.6	5.0	2.5	4.1	4.6	<0.05*
35–49	11.4	8.4	10.1	8.5	9.6	0.34
≥50	24.5	17.3	17.1	11.1	17.1	0.05
**All Ages**	7.7	6.0	5.6	6.0	6.3	<0.05*
<5	15.4	13.7	15.0	16.2	15.1	0.64
≥5	6.0	4.3	3.6	4.0	4.5	<0.05*

* Significant reductions in mortality rates over the years.

Males represented 55.1% of the total number of deaths. Overall mortality rates were significantly lower in females (5.7/1000 PYO) than in males (6.8/1000 PYO) (RR = 0.84 [95% CI: 0.73, 0.98], *P*<0.05). In persons ≥5 years old, females had significantly lower mortality rates (3.8 per 1000 PYO) than males (5.1 per 1000 PYO, RR = 0.75 [95% CI: 0.62, 0.91], *P*<0.05). Female infant mortality rates were 53.4 per 1000 PYO compared with 46.8 per 1000 PYO in male infants; however, this difference was not statistically significant, and there were no gender differences in child or overall under-five mortality rates.

Bi-weekly household visit data from the two weeks before death were available for 666 (89%) cases including 266 (40%) children under five years old and 400 (60%) persons ≥5 years old. There were 1996 controls for the 666 cases that had household morbidity data available. Among the cases 161 (61%) children under five years and 250 (63%) of persons aged >5 years reported at least one of the following cough, fever or diarrheal symptoms during the household visit, while among the controls 191 (24%) children under five years and 77 (6%) of persons aged >5 years reported at least one of the same symptoms. The percentages of cases and controls having fever were 27.8% and 4.6%. The corresponding percentages for cough were 28.8% and 8.0%, and 18.0% and 2.4% for diarrhea (Table 3). The percentage of cases and controls having both fever and cough were 16.7% and 3.3% respectively. The corresponding percentages for fever and diarrhea were 9.8% and 1.2%, and 9.9% and 1.6% for cough and diarrhea. We fitted a conditional logistic regression model for case/control status (death) including main effects for all three diseases and their three pair wise interaction terms. The 3-way interaction term was initially fit in the model and was non-significant, and thus subsequently dropped from the model. All three main effect variables for the diseases were significant (matched ORs = 11.4, 4.3, and 14.4 for fever, cough, and diarrhea, respectively) indicating that each disease is independently associated with death absent the other two (Table 4). The interaction terms for fever and cough (p<0.001) and for cough and diarrhea (*p* = 0.001) were both statistically significant and indicated that these two interactions were antagonistic (i.e. the risk factors reduce the effect of one another in each pair). The interaction term for fever and diarrhea was nonsignificant (*p* = 0.38). The matched ORs of death for fever and cough, fever and diarrhea, and cough and diarrhea versus no illness two weeks prior to death were 8.5, 105.4, and 11.9, respectively (Table 5). The matched OR of death with fever and cough versus cough alone was 2.0 (*p* = 0.015), but there was no evidence that deaths were more likely in persons with fever and cough versus fever alone. The matched OR of death was 9.3 for fever and diarrhea versus fever alone, and 7.3 for fever and diarrhea versus diarrhea alone. The matched OR of death for cough and diarrhea versus cough alone was 2.7 (*p* = 0.014) but there was no evidence that deaths were more likely in persons with cough and diarrhea than in persons with diarrhea alone.

**Table 3:** Distribution of reported symptoms by age among cases and controls two weeks before death in Kibera informal settlement, Kenya January 2007 to December 2010.

	Cough			Fever			Diarrhea		
Age in years	Cases (%)	Controls (%)	OR (95% CI)	Cases (%)	Controls (%)	OR (95% CI)	Cases (%)	Controls (%)	OR (95% CI)
<1	66 (47.1)	85 (20.2)	3.5 (2.3–5.3)	58 (41.4)	46 (11)	5.8 (3.6–9.1)	43 (30.7)	25 (6)	7.0 (4.1–12.0)
1–4	31 (24.6)	35 (9.3)	3.2 (1.9–5.5)	22 (17.5)	22 (5.8)	3.4 (1.8–6.4)	28 (22.2)	18 (4.8)	5.7 (3.0–10.8)
**<5**	**97 (36.5)**	**120 (15)**	**3.2 (2.4–4.4)**	**80 (30.1)**	**68 (8.5)**	**4.6 (3.2–6.6)**	**71 (26.7)**	**43 (5.4)**	**6.4 (4.2–9.6)**
5–9	4 (15.4)	4 (5.1)	3.4 (0.8–14.6)	8 (30.8)	3 (3.8)	11.1 (2.7–46.1)	2 (7.7)	1 (1.3)	6.4 (0.6–73.9)
10–17	3 (17.6)	1 (2)	10.7 (1.0–111.2)	6 (35.3)	0	NA	3 (17.6)	0	N/A
18–34	34 (18.7)	21 (3.8)	5.7 (3.2–10.2)	44 (24.2)	10 (1.8)	17.1 (8.4–34.8)	17 (9.3)	1 (0.2)	56.1 (7.4–424.9)
35–49	34 (27.9)	8 (2.2)	17.3 (7.7–38.7)	34 (27.9)	4 (1.1)	35.0 (12.1–101.1)	18 (14.8)	1 (0.3)	63.1 (8.3–478.5)
≥50	20 (37.7)	5 (3.2)	18.4 (6.4–52.6)	13 (24.5)	6 (3.8)	8.2 (2.9–22.9)	9 (17)	1 (0.6)	31.9 (3.9–258.7)
**≥5**	**95 (23.8)**	**39 (3.3)**	**9.2 (6.2–13.7)**	**105 (26.3)**	**23 (1.9)**	**18.2 (11.4–29.1)**	**49 (12.3)**	**4 (0.3)**	**41.7 (14.9–116.3)**
**All Ages**	**192 (28.8)**	**159 (8)**	**4.7 (3.7–5.9)**	**185 (27.8)**	**91 (4.6)**	**8.1 (6.1–10.7)**	**120 (18)**	**47 (2.4)**	**9.1 (6.4–13.2)**

**Table 4:** Assesment of independent association of symptom(s) with death stratified by age group using conditional logistic regression.

Age in years	Symptom	Matched Odds Ratio (95% Wald Confidence Limits)	P-value
<1	Fever	8.2 (2.7–24.7)	0.0002
	Cough	2.6 (1.4–5)	0.0041
	Diarrhea	8.9 (2.4–32.5)	0.0010
	Fever and Cough	0.2 (0.1–0.9)	0.0336
1–4	Cough	3.5 (1.5–8.1)	0.0032
	Diarrhea	8.1 (2.4–27.6)	0.0008
	Cough and Diarrhea	0.2 (0–0.8)	0.0238
**<5**	Fever	4.6 (2.2–9.6)	<.0001
	Cough	2.8 (1.7–4.6)	<.0001
	Diarrhea	8.1 (3.4–19.6)	<.0001
	Fever and Cough	0.3 (0.1–0.9)	0.0276
	Cough and Diarrhea	0.3 (0.1–0.8)	0.0210
5–9	Fever	13 (1.4–119.3)	0.0236
10–17	None		
18–34	Fever	64.3 (8.6–481.1)	<.0001
	Cough	3.3 (1.2–9)	0.0171
	Fever and Cough	0 (0–0.4)	0.0076
35–49	Fever	Undefined	0.0024
	Cough	12.7 (3–52.7)	0.0005
	Diarrhea	Undefined	0.0006
≥50	Cough	Undefined	0.0008
	Diarrhea	40.2 (2.1–783.2)	0.0148
≥5	Fever	26.4 (10.8–64.6)	<.0001
	Cough	7.2 (3.8–13.6)	<.0001
	Diarrhea	34.8 (9.5–127.6)	<.0001
	Fever and Cough	0.1 (0–0.3)	0.0002
**All Ages**	Fever	11.4 (6.7–19.4)	<.0001
	Cough	4.3 (2.9–6.4)	<.0001
	Diarrhea	14.4 (7.1–29.2)	<.0001
	Fever and Cough	0.2 (0.1–0.4)	<.0001
	Cough and Diarrhea	0.2 (0.1–0.5)	0.0010

**Table 5:** Conditional logistic regression model of death with reported symptom(s) as predictors.

Symptom(s)	Matched Odds Ratio (95% CI)	P-value
Fever, no cough, no diarrhea vs no illness	11.4 (6.7–19.4)	<.0001
Cough, no fever, no diarrhea vs no illness	4.3 (2.9–6.4)	<.0001
Diarrhea, no fever, no cough vs no illness	14.4 (7.1–29.2)	<.0001
Fever and cough, no diarrhea vs no illness	8.5 (5.5–13.1)	<.0001
Fever and diarrhea, no cough vs no illness	105.4 (32–346.8)	<.0001
Cough and diarrhea, no fever vs no illness	11.9 (5.6–25.3)	<.0001
Fever and cough, no diarrhea vs fever	0.7 (0.4–1.4)	0.3784
Fever and cough, no diarrhea vs cough	2 (1.1–3.4)	0.0151
Fever and diarrhea, no cough vs fever	9.3 (3–28.3)	<.0001
Fever and diarrhea, no cough vs diarrhea	7.3 (2.5–21.4)	0.0003
Cough and diarrhea, no fever vs cough	2.7 (1.2–6.1)	0.0140
Cough and diarrhea, no fever vs diarrhea	0.8 (0.3–2.1)	0.6890

Among sick children and persons aged ≥5years, health care outside the home during the two weeks before death was sought for 121 (75%) children under-five and for 167 (67%) persons aged ≥5years. Male children <5 years old with cough or diarrhea or fever noted during the two weeks before death were slightly less likely (74%) to seek care at a health facility than females <5 years old with the same symptoms (77%) (OR = 0.86, [95% CI: 0.39–1.87]). Similarly, for persons ≥5 years of age, males with cough, diarrhea or fever were less likely (63%) to seek care than females (72%) (OR = 0.68, [95% CI: 0.39–1.20]). However, the differences were not statistically significant.

## Discussion

The findings of our study indicate that essentially 5% (50 deaths per 1000 child-years) of infants die in the first year of life in this urban slum setting. In contrast with rural areas, where clinical services are often hard to access [14], six licensed health facilities within the surveillance villages are available in close proximity, and yet are often not timely utilized, even with illnesses serious enough to result in death [13]. This suggests that more work is needed to characterize health conditions in urban settings and understand health utilization determinants.

Comparison of illnesses among people living in this slum area and a sparsely populated rural in western Kenya suggest that rates of cough, febrile illness and diarrheal disease can be quite dissimilar [15]. Some etiologic agents for these syndromes appear to be disparate as well [16], [17]. Effective programs and interventions to improve health outcomes in urban slum settings may need to be configured quite differently than in rural areas. This is becoming an increasingly important issue as urbanization in Kenya continues to occur at approximately 7% annually [18] with people migrating from rural areas nearly universally into densely populated urban slums where sanitation, hygiene, and water supplies are sub-optimal; in 2012, >60% of the Nairobi population resided in urban slums [19]. UNICEF's 2012 State of the World's Children Report emphasized that even where services are nearby, children growing up in poor urban settings face significant health risks [9].

The findings from this study suggest that mortality rates in the Nairobi urban slums were at par or higher than rates reported from an urban slum in India (6.2 per 1000 PYO) [10]. Data analyzed from 22 countries indicated that only five countries (Namibia, Eritrea, Madagascar, Uganda and Malawi) recorded declines in urban child mortality in line with the MDG target of about 4% per annum [20]. Although the Kenya Demographic and Health Survey from 2008 reported a decline in under-five mortality rates, continued urban migration without either infrastructural improvements or programs targeting unique urban health problems, may impede and potentially reverse progress in reducing childhood mortality. Consistent with that concern, the Nairobi Cross-sectional Slum Survey (NCSS), carried out by the African Population and Health Research Center (APHRC) in 2000 showed that urban under-five mortality has increased over time, and suggested that urban poor children have higher mortality than their rural counterparts [6]. The surveillance platform which provided data for this study includes free, high quality health care for acute illnesses for all surveillance participants. We believe that availability of high quality and free health services offered by the study clinic has impacted severe health outcomes; we are unable to confirm this because we do not have baseline disease incidence or mortality rates from the surveillance area before the program began. However, over the course of the study, there was a significant reduction in mortality rate among persons 18–34 years old. While free prevention services and treatment of children in urban areas are relatively ubiquitous, access for good quality and free care for adults is less common. We speculate that the introduction of free, high quality health care to residents within the surveillance community may have had differential benefits for adults, who otherwise had limited health utilization options. Further study will be needed to explore this potential benefit of health systems strengthening within impoverished informal settlements on severe illness and disease outcomes.

A limitation of this study is that the presence of the study clinic within the surveillance area may have led to underestimation of mortality rates for the majority of urban slum settings where a similar option does not exist. Also, it is difficult to compare our findings with most other studies providing mortality rates. Most childhood mortality calculations use the number of live births as the denominator. PBIDS did not collect data on births during the study period; however, since we conducted longitudinal surveillance within an area with known population size, we were able to use a rigorously defined denominator of people under surveillance to provide mortality rates per person years of observation. Nonetheless, it is likely that mortality rates were underestimated for very young children, early in infancy, since some (especially neonates) may die before they are officially enrolled into the system. While it was not possible to characterize mortality in the context of live-births, the description of the proportion of the population with death outcomes annually provides capacity to estimate overall raw numbers of deaths in similar settings where population size is known, and, therefore, to estimate the potential impact of interventions with known effectiveness on numbers of deaths averted.

We assumed that symptoms experienced two weeks prior death were associated with the death; however, the degree that this was indeed true was likely impacted by the timing of the household visit. Despite differences observed between cases and controls, it is likely that in many cases, symptoms reported during the biweekly visit were unrelated to the illness or event causing death. Formal verbal autopsies and pathologic studies are needed to shed more light on likely causes of death in this setting.

PBIDS provided an accurate denominator for calculating mortality rates across different age groups. The results of this analysis suggest that although health care for acute illnesses is accessible, high proportion of deaths may be associated with infectious disease symptoms, and these illnesses do not appear to have led to clinic visits.To derive well-targeted and effective interventions, further research among urban slum dwellers is needed to provide cause specific mortality rates and determinants, as well as data of impact of targeted public health interventions in similar settings.
